# Supplementary material for: Domain wall magnetic tunnel junction-based artificial synapses and neurons for all-spin neuromorphic hardware
Source: Nat Commun. 2024 May 28;15:4534. doi: 10.1038/s41467-024-48631-4 (PMC11133408; doi:10.1038/s41467-024-48631-4)
Supplement: Supplementary file 1 — Supplementary Information [file 41467_2024_48631_MOESM1_ESM.pdf]

Table of Contents:

Figure S1 Crystal structure and spin configuration of W/CoFe(001) structure employed for DMI calculations.

Figure S2 The approaches of improving TMR and alleviating the DW motion stochasticity from high quality films stack growth and optimized fabrication process.

Figure S3 TMR mapping from a 10×10 EBL and a UV-lithography patterned MTJs array.

Figure S4 TEM images and EDS mapping results of a pinning center.

Figure S5. HRTEM images and investigation results of PC edge transition position.

Figure S6 DW pinning reliability characteristics.

Figure S7 Extensive simulation of prototype sigmoidal activation function generator.

Figure S8 Experiment setup of typical array consisting of all-spin synapse and activation function generator.

Figure S9 Benchmark of latest representative sigmoid activation function generators.

Figure S10 Dilemma of simultaneous electrical write and detection in the present DW-MTJ based synapse and neuron.

Table S1 Micro-magnetic simulation parameters.

Table S2 Cadence simulation parameters.

Note S1 Device fabrication process optimization.

Note S2 Characterization of pinning center.

Note S3 Verilog-A model of the proposed sigmoid activation function generator.

Note S4 Performance evaluation of sigmoid activation function generator.

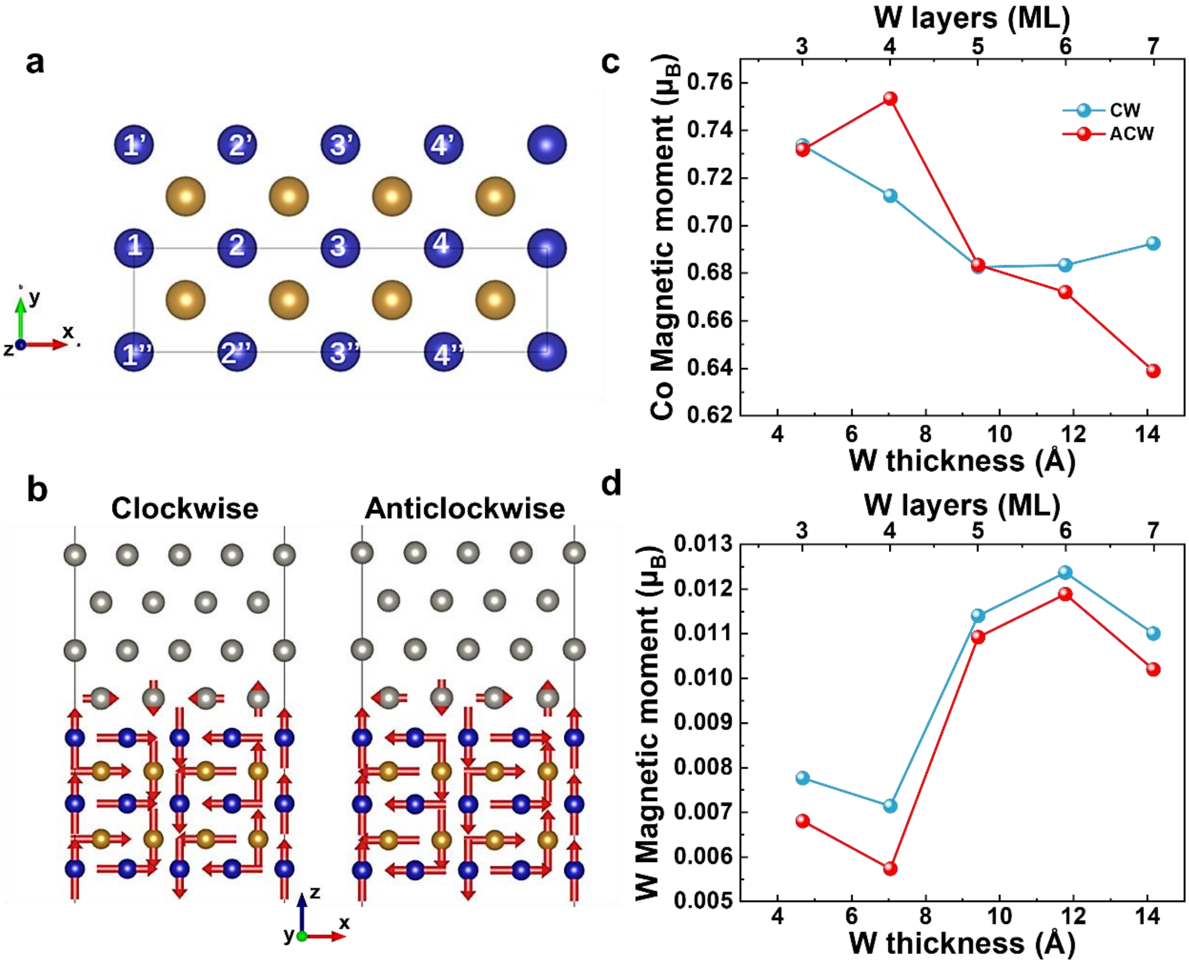

Figure S1. The crystal structure used for DMI calculation. **a** Top view of CoFe supercell and **b** side view of bcc W (001)/CoFe (001) structure with clockwise and anticlockwise spin configurations. **c** magnetic moment of Co atom in the two spin configurations as a function of W thickness. **d** induced magnetic moment of W with opposite spin configuration as a function of W thickness. Spin moments of Fe atoms are multiplied by 10 for demonstration.

The structure of W/CFB structure modelled here for DMI calculation is demonstrated in **Figure S1**. 3-7 monolayer (ML) thick body-centered cubic (bcc) W (001) coats on CoFe (001) layer. The gray, blue and yellow symbols correspond to W, Co, Fe atoms, respectively. Referring to equation (1) in the main text, the microscopic DMI strength is extracted by calculating the difference between the DFT energy E_{CW} and E_{ACW} of clockwise and anticlockwise spin spirals indicated by the red arrows. For instance, the energy of atom 2, as shown in **Figure S1a**, can be written as,

$$E_2 = \frac{1}{2}[\overrightarrow{d_{23}} \cdot (\overrightarrow{S_2} \times \overrightarrow{S_3})] + \frac{1}{2}[\overrightarrow{d_{21}} \cdot (\overrightarrow{S_2} \times \overrightarrow{S_1})] + \frac{1}{2}[\overrightarrow{d_{22'}} \cdot (\overrightarrow{S_2} \times \overrightarrow{S_{2'}})] + \frac{1}{2}[\overrightarrow{d_{22''}} \cdot (\overrightarrow{S_2} \times \overrightarrow{S_{2''}})] + E_{other} \quad \text{(S1)}$$

Where 1/2 suggests energy sharing between the two atoms of each pair, and the E_{other} denotes to energy contributions consist of spin independent, anisotropy and symmetric exchange terms. Because that $\overrightarrow{S_2}$ is parallel to $\overrightarrow{S_{2'}}$ and $\overrightarrow{S_{2''}}$, equation (S1) can be simplified,

$$E_2 = \frac{1}{2}[\overrightarrow{d_{23}} - \overrightarrow{d_{21}}] + E_{other} \quad \text{(S2)}$$

The energy of atom 2 for opposite spin configuration shown in **Figure S1b** written as,

$$E_{2,CW} = d_{12}^{y} + E_{other} \quad \text{(S3)}$$

$$E_{2,ACW} = -d_{12}^{y} + E_{other} \quad \text{(S4)}$$

Therefore, the total DMI energy in the cell and the value per bond are determined,

$$\Delta E_{DMI} = (E_{CW} - E_{ACW}) = 8d_{12}^{y} \quad \text{(S5)}$$

Therefore, the parameter m in equation (1), which depends on the wavelength of the cycloid, is derived as 8 in our structure.

The magnetic moment of Co and induced magnetic moment of W with two spin configuration is plotted in **Figures S1c** and **S1d** as a function of W thickness, respectively. No straightforward and obvious correlation between magnetic moment and W thickness can be extracted. Consequently, the magnetic moment could not account for the W thickness dependence of DMI.

Note S1: Device fabrication process optimization.

The Hall bar devices of 15 μm width and 50 μm length, synaptic devices with 2 μm × 50 μm channel and 50 μm × 50 μm nucleation pads and neural devices with 2 μm × 75 μm channel and 50 μm × 50 μm nucleation pads were fabricated using photolithography and ion milling, followed Ti(20 nm)/Au(80 nm) electrodes. By employing MagVision system with a probe station, a constant DC of 0.1 μA was applied on the MTJ to read out the junction voltage drop during the H_z scan in the TMR measurement for the MTJ device, whereas in the *R-H* loops measurement of synaptic and neuron devices, a 300 μA read DC was applied for junction resistance reading.

To improve low TMR issue and alleviate the DW motion stochasticity issue in the present work, a lot of works have been done to optimize the device fabrication process as shown in

Figure S2.

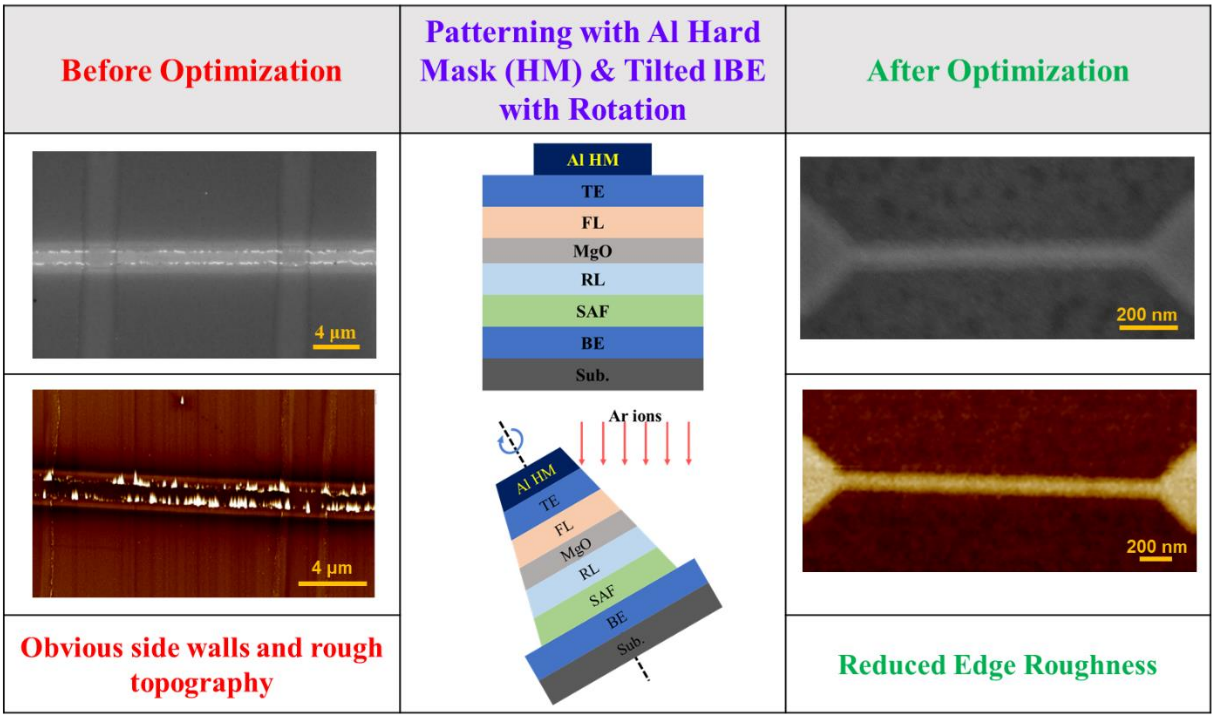

Figure S2. Typical SEM and AFM images data with the illustration of improved side-wall topology, morphology, and edge roughness, after devices fabrication engineering by using Al hard-mask (HM) and tilted ion-beam etching (IBE) process optimization.

The film surface/interface disorder and the roughness of device edge can be improved by optimizing the film deposition technology and device fabrication process[1-4]. In our work, besides securing the high-quality epitaxial growth of the films stack with post annealing treatment to reduce the intrinsic disorders and pinning effectively, the dedicated devices fabrication processes (i.e., the improved lithography with Al hard-mask and the optimized ion-beam etching with 30º tilting angle and rotation) were systematically executed to reduce the edge roughness and to minimize the imperfection sites, as verified by below tabulated extensive SEM and AFM scanning images data with illustration of improved side-wall topology and edge roughness.

The TMR distribution map of our optimized 10×10 MTJ arrays with dimension of 100 nm in diameter for EBL device and 50 μm in length×2 μm in width for UV-lithography device is shown in **Figure S3**. It suggests that the TMR is improved markedly by the fabrication process optimization. Especially for the UV-lithography one, a more uniform and considerable TMR distribution map is demonstrated. However, for the EBL one, the uniformity and yield rate must improve further in our future work.

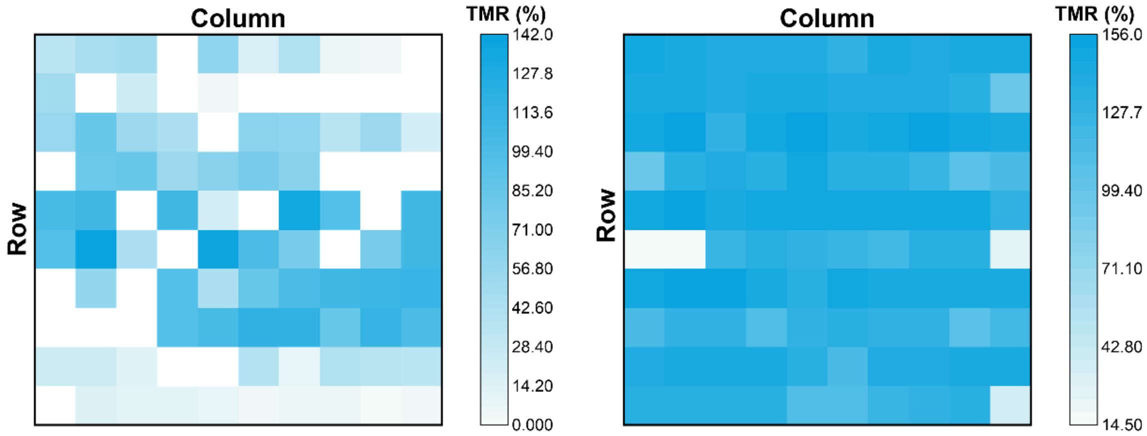

Figure S3. TMR mappings from a 10×10 EBL (left panel) and UV-lithography (right panel) patterned MTJ devices array with dimension of 100 nm in diameter and 50 μm×2 μm, respectively.

Note S2: Characterizations and investigations of pinning center

The pinning centers are designed by selectively etch on the SOC layer according to the SOC layer thickness dependence of DMI which is approved in **Figures 1** and **2** in the main text. To visualize etch-induced W thickness modulation, the TEM is performed and the results of etched PC is shown in **Figure S4**. As shown in **Figure S4a,** two positions marked as Position 1 and Position 2, referring to unetched PC edge and etched PC pit bottom respectively, is picked and highlighted by the orange dashed box. EDS mapping and line scan EDS intensities results are presented in **Figures S4b** and **S4c.** For unetched position 1 (upper panel), strong signals of Ta and Ru are observed both in EDS mapping and line scan results. While there is no corresponding obvious Ta and Ru signals for Position 2 (lower panel), and the W thickness is reduced approximately 1 nm compared with that thickness of Position 1 due to the ion beam milling. From these TEM results, we could conclude that the W layer is partially etched in the PC region by IBE, which anticipates a higher DMI in the PC according to W thickness-DMI relation.

The high-resolution TEM (HRTEM) results of PC edge transition position are demonstrated in **Figure S5**. Obviously, the thickness of Ta capping layer reduced gently from the PC edge to PC center and the Ru layer is almost unetched, consistent with the EDS results in **Figure S4**.

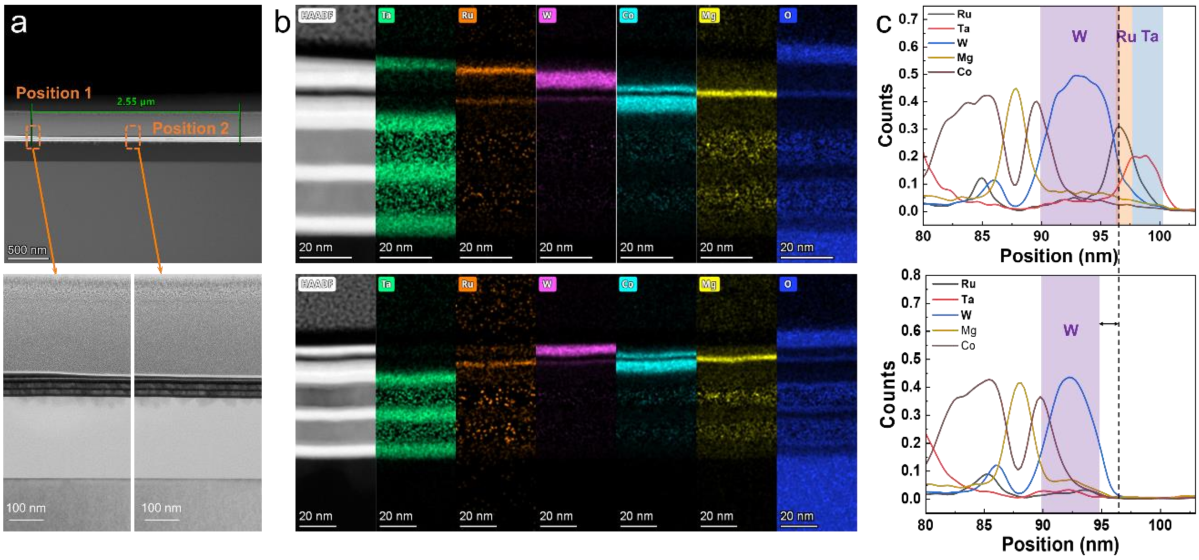

Figure S4. TEM images and EDS mapping results of a pinning center. **a** TEM images of the etched PC with a designed width of 3 μm (upper panel) and enlarged images of Position 1 and Position 2 (lower panel) highlighted by orange dashed box, which denote to the unetched PC edge and etched PC pit bottom, respectively. **b** EDS mapping results of Position 1 (upper panel) and Position 2 (lower panel) obtained from the HADDF image. **c** line scan EDS intensities of Ru, Ta, W, Mg, Co of Position 1 (upper panel) and Position 2 (lower panel).

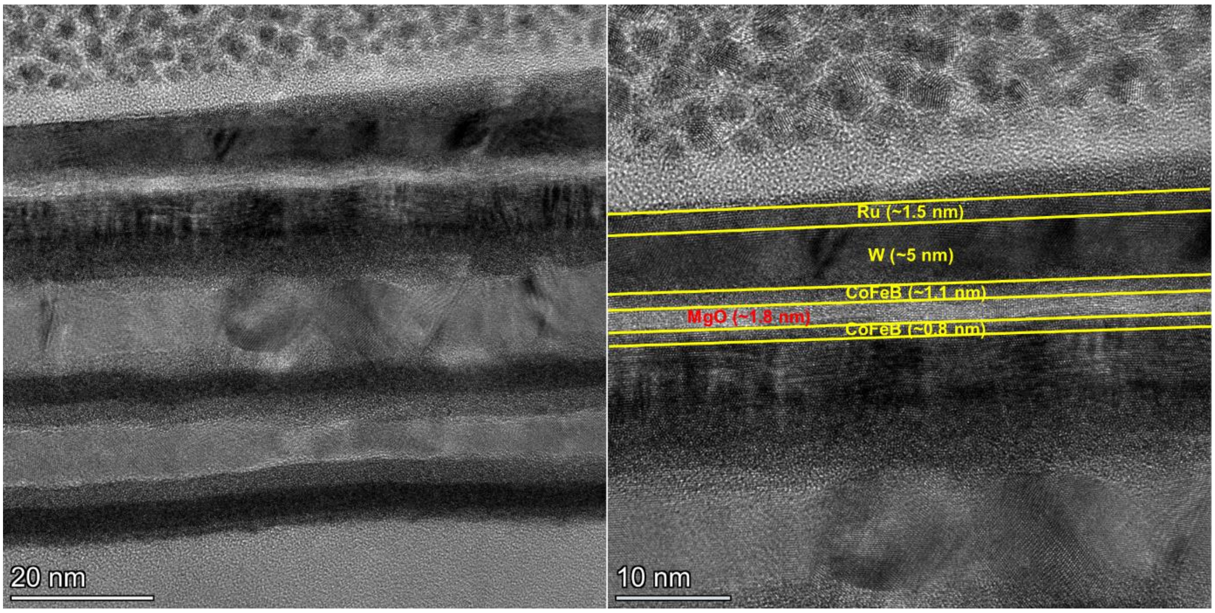

Figure S5. HRTEM images and investigation results of PC edge transition position.

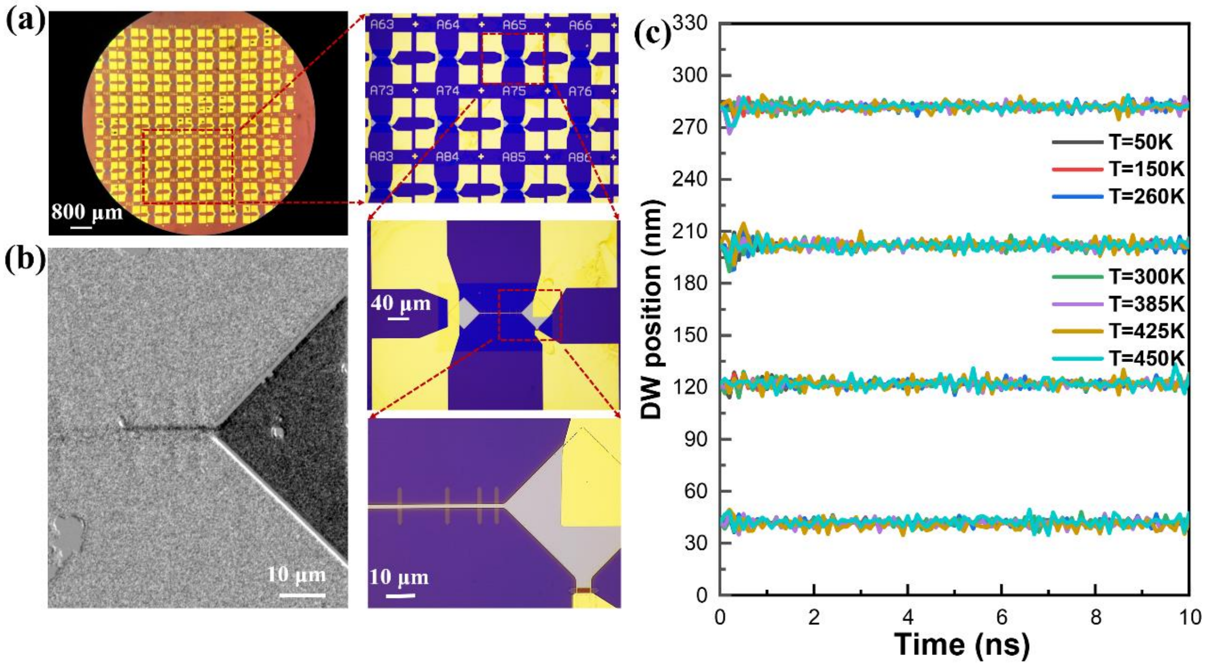

Figure S6. (a) and (b) Optical microscope images of SOT-DMI DW devices array with non-uniformly patterned pinning centers that further corroborate the *i*DMI induced pinning mechanism rather than the sources such as line edge roughness and magneto crystalline defects. (c) Corresponding polar-MOKE image of DW pinning state collected after a time-span longer than 10 months since fabrication. (f) DW pinning behaviors in the pinning center under different temperature of 50 K, 150 K, 260 K, 300 K, 385 K, 425 K and 450 K.

Furthermore, the DW can be pinned reliably with a robust energy barrier at room temperature after a time-span longer 10 months since fabrication, further corroborating that the iDMI induced pinning mechanism rather than the sources such as line edge roughness and magneto crystalline defects. Notably, the DW was pinned reliably with robust energy barrier E_B at room temperature as shown in **Figures S6a** and **S6b**. Furthermore, by using the same micromagnetic simulation parameters as original manuscript work, the extensive temperature-dependent DW pinning stability results also demonstrate the stabilized pinning characteristics under a wide temperature range of 50~450 K (**Figure S6c**). Clearly, the DW is stabilized in the pinning centers without any depinning collapse when the temperature is up to 450 K (i.e., 150 ^{o}C) that is aligned with the generic industrial criteria of Product Reliability Testing.

Supplementary Table S1. Micro-magnetic simulation parameters of 14-state sigmoid activation function generator.

Parameters	Description	Values	
M_s	Saturation magnetization	1×10^6 A/m	
K_u	Magnetic anisotropy	1.3×10^6 J/m^3	
A_{ex}	Exchange stiffness constant	1×10^{-11} J/m	
α	Gilbert damping constant	0.02	
θ_{SH}	Spin hall angle of SOC layer	-0.35	
t_W	Pulse width	0.5 ns	
t_p	Pulse period	4 ns	
J_e	Current density	5.5×10^7 A/cm^2	
$(L\times W\times t)_{FL}$	Free layer size	Free layer1	276 nm×50 nm×1.2 nm
		Free layer2	256 nm×50 nm×1.2 nm
		Free layer3	197 nm×50 nm×1.2 nm
		Free layer4	131 nm×50 nm×1.2 nm
		Free layer5	76 nm×50 nm×1.2 nm
		Free layer6	38 nm×50 nm×1.2 nm
		Free layer7	14 nm×50 nm×1.2 nm
$(L\times W\times t)_{PC}$	Pinning center size	20 nm×50 nm×1.2 nm	
$(L\times W\times t)_{Reservoir}$	DW reservoir source size	10×50 nm×1.2 nm	
D_{PC}	DM exchange constant of PCs	1 mJ/m^2	
D_{FL}	DM exchange constant of free layer	Free layer1	0.75 mJ/m^2
		Free layer2	0.65 mJ/m^2
		Free layer3	0.24 mJ/m^2
		Free layer4	0.39 mJ/m^2
		Free layer5	0.29 mJ/m^2
		Free layer6	0.24 mJ/m^2
		Free layer7	0.18 mJ/m^2

Micromagnetic simulations were performed to verify the feasibility of the proposed sigmoidal activation function generator (14-states) using an open-source tool MuMax3 (Ref. 5). Detailed simulation parameters are listed in **Table S1**. The magnetization of the two ends of the nanowire is pinned by antiferromagnet in the opposite direction for DW nucleation[6]. After the system relaxes with fully energy minimization, a "↑→↓" DW forms and gets pinned in the PC adjacent to the DW nucleation region. Then a series of SOT current pulses, 0.5 ns in width, 4 ns in duration, and 5.5×10^7 A/cm^2 in density amplitude, corresponding to an energy consumption of 173.9 fJ per pulse, is applied to drive the DW motion state-by-state. The DW dynamics are

governed by the Landau–Lifshitz–Gilbert (LLG) equation[7],

$$\frac{d\hat{m}}{dt} = -\gamma\hat{m}\times H_{eff} + \alpha\hat{m}\times\frac{d\hat{m}}{dt} + \gamma\tau_{\mathrm{SOT}}\hat{m}\times\left(\hat{m}\times\hat{y}\right) \quad \text{(S6)}$$

where γ and α are the gyromagnetic ratio and the Gilbert damping constant, respectively, $\hat{m}$ is the unit magnetization vector, and $\hat{y}$ denotes spin polarization direction. The field-like SOT torque is neglected for simplicity. $\tau_{\mathrm{SOT}} = \left(\hbar/2e\right)\left(\theta_{\mathrm{SH}} / M_S t_{FL}\right) J_e$ describes the magnitude of the anti-damping-like SOT. In this case, H_{eff} is considered as[7],

$$H_{eff} = \frac{2A}{M_S}\frac{\partial^2\hat{m}}{\partial x^2} + \frac{2K}{M_S}m_z\hat{z} + \frac{2K_d}{M_S}m_y\hat{y} - \frac{2D}{M_S}\left(\hat{y}\times\frac{\partial\hat{m}}{\partial x}\right) \quad \text{(S7)}$$

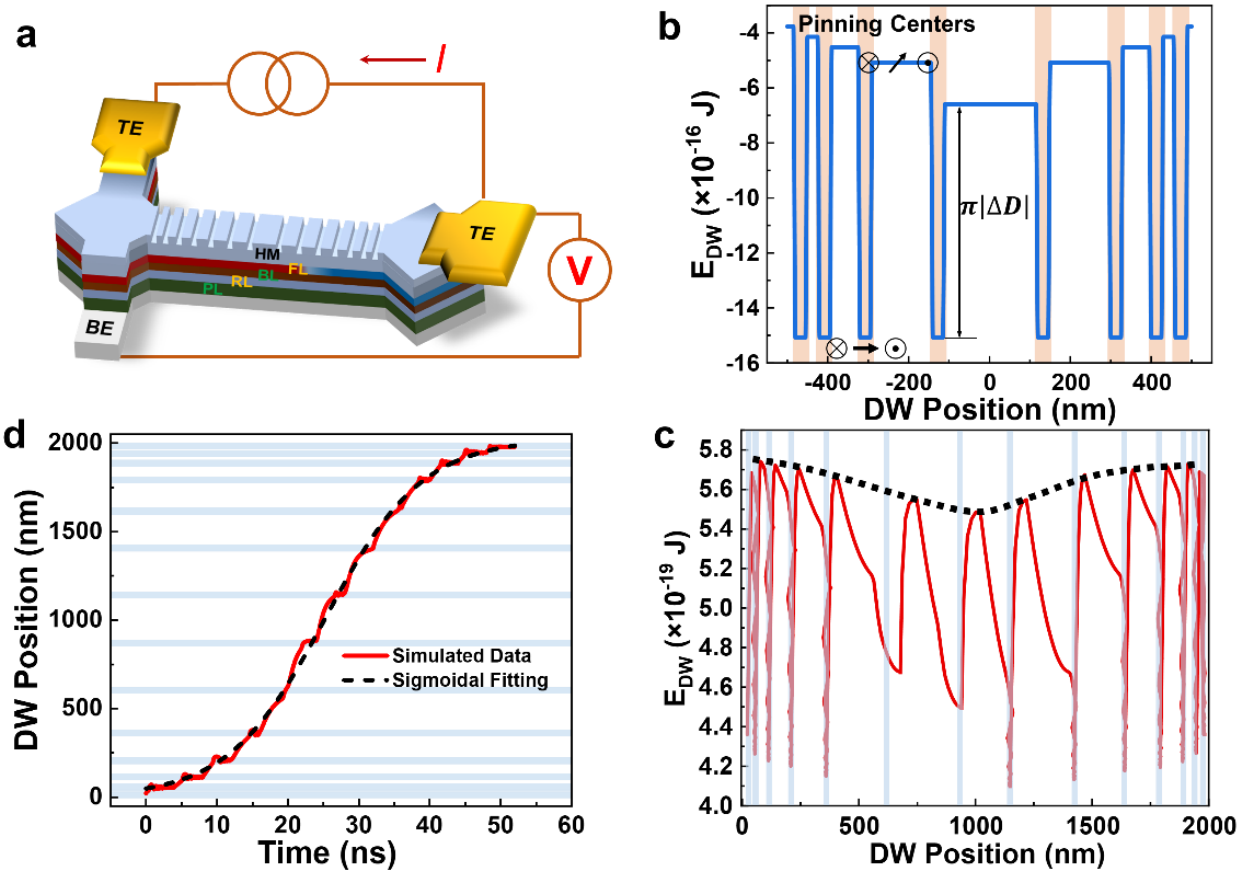

Figure S7. Prototype sigmoidal activation function generator. a Schematic diagram of proposed spintronic neuron based on DW-pMTJ. **b** DW energy distribution as a function of DW position with different *i*DMI according to *q*-*φ* model. Regions in pink rectangles denote PCs with larger DMI constant. Micromagnetic simulation results: **c** DW energy distribution as a function of DW position, and **d** DW position under a sequential SOT current pulse. Regions in blue rectangles refer to PCs.

As demonstrated in **Figure S7a**, the PCs is non-uniformly distributed based on piecewise approximation. The DW energy distribution versus DW position based on *q*-*φ* model in **Figure**

S7b explains the origin of nonlinearity in the sigmoidal activation function generator. The designed PCs, marked by pink regions in **Figure S7b** with greater DMI correspond to an energy potential well with the barrier height of $\pi|\Delta D|$. In our design, all PCs shares the same DMI constant, while the DMI constant of the nanowire segments separated by the PCs is related to the segment length. The designed DMI constant distribution can be further explained by the IBE-induced stress effects, as reported by several research work[8]. With the designed DMI constant distribution, the energy barrier height is nonuniformly distributed along the DW motion direction as shown in **Figure S7b**.

The DW position dependent DW energy was further analyzed by micromagnetic simulation as shown in **Figure S7c**, which is in line with **Figure S7c**. A non-linear distributed barrier height is observed as indicated by the black dash line, accounting for the non-linear DW motion distance in same duration. The results in **Figure S7d** are well fitted with a shifted sigmoid function as illustrated by the black dashed lines. The fitting results demonstrate that the profile of DW position with corresponding MTJ magneto-resistance analogs a sigmoidal function of time or the pulse number. It can also be a function of pulse amplitude or pulse width analytically. These simulations can provide insights into the intricate dynamics of the magnetic domain wall and its interaction with the pinning centers, leading to the observed nonlinearity and the generation of the activation function.

The actual experiment system is demonstrated in **Figure S8a**, with every module labeled by a white tag. A Labview program in host computer was used to control timing sequence with synchronizing all-spin NC hardware with integration of CMOS components. First, command sent from HC to MCU on board triggers pre-neuron signals at the I/O ports connected to 5 V power supply through pull-up resistors. The pre-neuron signals are applied to switch on the transistors connected in series with SOT-MTJ synapses in the peripheral circuits. The SOT-MTJ synapses were connected in series with a reference resistor with intermediate resistance constituting a voltage divider with read voltage V_{Read}. The node voltage was fed into operational amplifier with bipolar power supply (V_{cc}, V_{ee}), and compared to a reference voltage V_{Ref} subsequently, to generate a output pulse consequently to switch on the transistor linking current pulse generator and neuron devices.

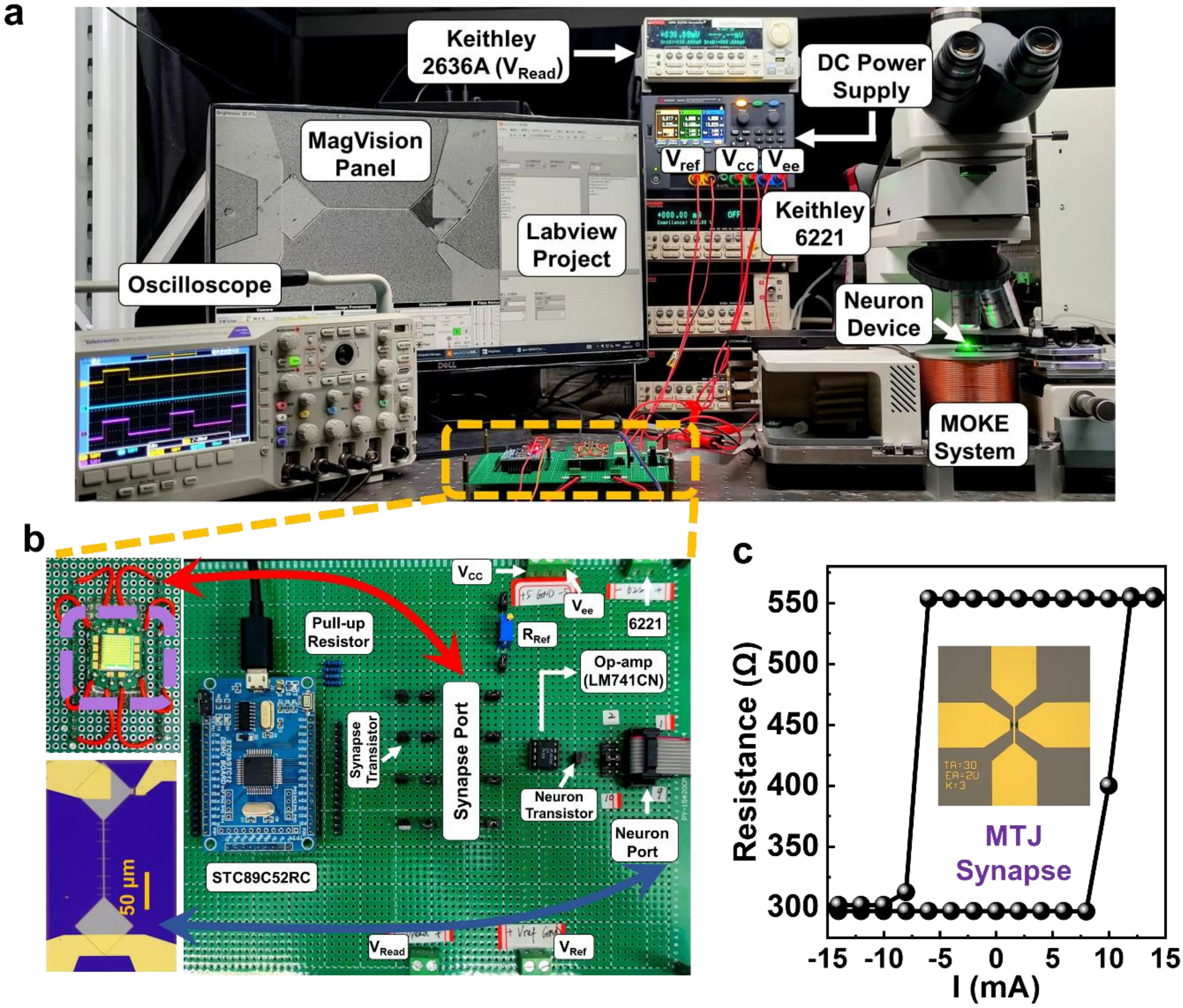

Figure S8. **a** Photograph of the whole experiment system. **b** Hardware implementation of one partial array in **Fig. 7 a** with 4 binary SOT-MTJ as synapse and 1 proposed neuron device. **c** R-I loop of the SOT-MTJ used as synapse in **b**.

The core component, a universal board with MCU and peripheral circuits, is marked by orange dashed box in **Figure S8a**, the magnified view of which is shown in **Figure S8b** too. The MCU serves as input layer to output pre-neuron signals in sequence. Four SOT-MTJs are connected to a PCB carrier by wire bonding to serve as synapses, highlighted by purple dashed box in **Figure S8b**. The PCB carrier is then attached to another universal board by jump wire, which is connected to other peripheral circuits by pin headers. A typical SOT switching loop of the SOT-MTJ synapse is shown in **Figure S8c**, with R_P, R_{AP} of 296, 557 Ω respectively, corresponding to a TMR of 88%. All SOT-MTJ synapses were set to R_{AP} state at begin by SOT current pulse with 5 μs pulse width and 15 mA pulse amplitude at begin. The SOT-MTJ synapses

are connected in series with a reference resistor with intermediate resistance approximately of 426 Ω ($\frac{R_P + R_{AP}}{2}$) constituting a voltage divider with read voltage V_{Read} of 30 mV.

During testing process , command sent from host computer to MCU on board triggers pre-neuron signals at the I/O ports connected to 5 V power supply through pull-up resistors firstly. The pre-neuron signals are applied to switch on the read control transistors connected in series with SOT-MTJ synapse then. The node voltage is fed into an operational amplifier with bipolar power supply (V_{cc} 5 V, V_{ee} -5 V), and compared to a reference voltage V_{Ref} of 16 mV subsequently, to generate a output pulse consequently to switch on the write control transistor linking current pulse generator and neuron device. With appropriate delay, a pulse command from host computer is sent to Keithley 6221 to trigger a current pulse (20 μs in pulse width) applied on write channel to switch state of neuron device. The MOKE system, synchronously controlled by the Labview program on HC through communication with MagVision interface starts to capture Kerr images after the current pulse applied. The modules are not strictly synchronized. Therefore, a prolonged pre-neuron signal is utilized for effective demonstration on purpose.

Supplementary Table S2 Cadence simulation parameters.

**Symbol**	**Description**	**Values**
Node	Technology node	28 nm
V_{DD}	Supply Voltage	1.2 V
L_{MOS}	Channel length of MOSFET transistor	30 nm
W_{MOS}	Channel width of MOSFET transistor	100 nm
TMR	Tunnel magnetoresistance ratio	150%
$(L\times W\times t)_{HM}$	Heavy metal size	2 μm×50 nm×5 nm
ρ_{HM}	Resistivity of heavy metal	230×10^{-8} Ω·m
t_{ox}	Thickness of barrier layer	1.2 nm
RA	Resistance-Area-Product	80 Ω·μm^2

Note S3: Verilog-A model of proposed sigmoidal activation function generator.

The Verilog-A model of proposed sigmoidal activation function generator is referring to a 1-D q-φ-χ-Δ model[9,10]. This model describes the DW in terms of three time-varying coordinates: q(t), Φ(t), χ(t) corresponding to the position of DW center on the *x*-axis, the angle of internal DW magnetization (m_{DW}), the angle of DW normal plane (n_{DW}) with the *x*-axis. These three coordinates have the following relationship:

$$(1+\alpha^2)\ \dot{q}=\frac{\Delta}{\cos\chi}(\Omega_A+\alpha\Omega_B) \tag{S8}$$

$$(1+\alpha^2)\ \dot{\Phi}=-\alpha\Omega_A+\Omega_B \tag{S9}$$

$$\dot{\chi}=\frac{(\frac{6\gamma_0}{\alpha\mu_0 M_s\Delta\pi^2})}{\tan^2\chi+(\frac{L_y}{\pi\Delta\cos\chi})^2}[-\sigma\sin\chi+\pi DQ\sin(\Phi-\chi)-\mu_0 H_K M_s\Delta\sin[2(\Phi-\chi)]] \tag{S10}$$

Where Ω_A and Ω_B are given by

$$\Omega_A=-\frac{1}{2}\gamma_0 H_K\sin[2(\Phi-\chi)]-\frac{\pi}{2}\gamma_0 H_y\cos\Phi+\frac{\pi}{2}\gamma_0 H_x\sin\Phi+\frac{\pi}{2}\gamma_0 H_{DMI}Q\sin(\Phi-\chi) \tag{S11}$$

$$\Omega_B=\gamma_0 QH+\frac{\pi}{2}\gamma_0 QH_{SH}\cos\Phi \tag{S12}$$

The H_{SH} and H_{DMI} refer to anti-damping SOT effective filed and DMI effective field respectively. The total field $H=H_z+H_p(X)$ include applied magnetic field along z-axis and spatial-dependent pinning field

$$H_p(X)=-\frac{1}{2\mu_0 M_s L_y L_z}\frac{\partial V_{pin}(X)}{\partial X}. \tag{S13}$$

The parallel resistance of MTJ can be expressed by Brinkman model:

$$R_P=\frac{t_{ox}}{FA\sqrt{\varphi_{ox}}WL}e^{\left(\frac{2\sqrt{2m|e|\varphi_{ox}}t_{ox}}{\hbar}\right)} \tag{S14}$$

where φ_{ox} is the potential barrier height of MgO and m is the electron mass. FA is a fitting coefficient, calculated by $FA=3.3141\times10^{-7}/RA$, where RA is the resistance–area product.

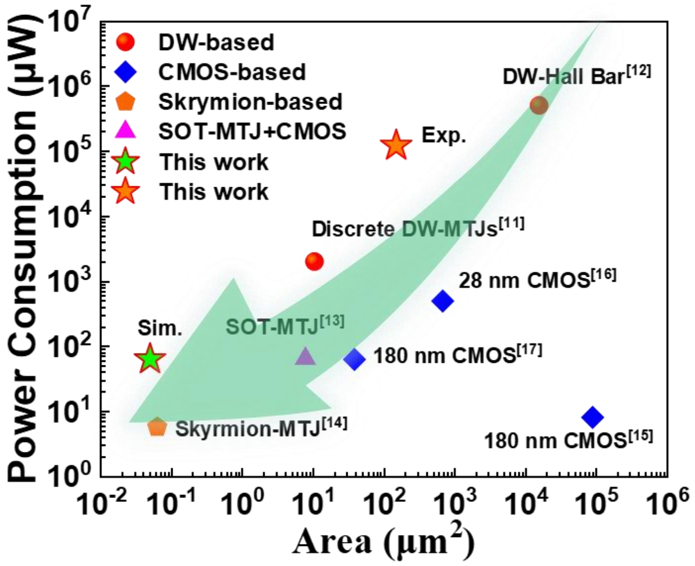

Figure S9 benchmark of latest representative sigmoidal activation function generator[11-17].

Note S4: Performance evaluation of sigmoid activation function generator.

To comprehensively demonstrate the performance of the device, we have listed the calculations of power and energy consumption of our device and the data extracted from other highly relevant reports.

Discrete DW-MTJ[11]

$$P = I^2R = (J_e wh)^2 R \approx (7\times10^{10}\, A/m^2 \times 1.6\times10^{-6} m \times 6\times10^{-9} m)^2 \times 5.2\times10^3 \Omega \approx 2.3\ mW$$

$$E = P\times t_p = 2.3\times10^{-3} W \times 8\times10^{-9} s = 18.4\ pJ$$

*The reset operation is realized through external magnetic field, so only the energy consumption of set operation is estimated. The reset operation is realized through external magnetic field, so only the energy consumption of set operation is estimated. w, h, ρ, l, corresponds to width, thickness, resistivity, and length of SOC layer, respectively.

DW-hall bar[12]

$$P = IV = (J_e wh)V \approx 2\times(1\times10^{11} A/m^2 \times 60\times10^{-6} m \times 4.2\times10^{-9} m)\times 20V \approx 1\ W$$

$$E = P\times t_p = 1W \times 500\times10^{-3} s = 0.5\ J$$

Skyrmion MTJ[14]

$Set:$

$$P = I^2 R = (J_e wh)^2 \frac{\rho l}{wh} = J_e^2 wh\rho l = (4.12\times10^{10} A/m^2)^2 \times 60\times10^{-9} m \times 2\times10^{-9} m \times 200\times10^{-8} \Omega\cdot m \times 150\times10^{-9} m = 0.061\ \mu W$$

$$E = P\times t_p = 0.061\times10^{-6} W \times 2\times10^{-9} s \times 7 = 0.85\ fJ$$

$\mathrm{Re}\,set:$

$$P = I^2 R = (J_e wh)^2 \frac{\rho l}{wh} = J_e^2 wh\rho l = (1.5\times10^{11} A/m^2)^2 \times 60\times10^{-9} m \times 2\times10^{-9} m \times 200\times10^{-8} \Omega\cdot m \times 150\times10^{-9} m = 0.81\ \mu W$$

$$E = P\times t_p = 0.81\times10^{-6} W \times 0.5\times10^{-9} s \times 7 = 3.24\ \mathrm{fJ}$$

This Work (8-state Experiment)

$$P = I^2 R = (0.11A)^2 \times 1\times10^3 \Omega = 0.121\ W$$

$$E_{single} = P\times t_p = 0.121W \times 10\times10^{-6} s = 1.121\ \mu J / Pulse$$

$$E_{total} = (P\times t_p)\times n = 0.121W \times 10\times10^{-6} s \times 14 = 16.94\ \mu J / Operation$$

$$f = 1/(n\times t_p) = 1/(14\times10\times10^{-6} s) = 7.14 KHz$$

where n refers to the total pulse number in an operation comprising of set and reset process. Both of the set and reset operation is implemented by state-by-state DW motion driven by a series of identical pulse. Therefore, for a 8-state device, $n = 2\times7 = 14$.

This Work (8-state Simulations)

$$P = I^2 R = (J_e wh)^2 \frac{\rho l}{wh} = J_e^2 wh\rho l = (0.33\times10^{12} A/m^2)^2 \times 50\times10^{-9} m \times 5\times10^{-9} m \times 230\times10^{-8} \Omega\cdot m \times 1\times10^{-6} m = 62.6\ \mu W$$

$$E_{single} = P\times t_p = 62.6\times10^{-6} W \times 580\times10^{-12} s = 36.3\ fJ / Pulse$$

$$E_{total} = (P\times t_p)\times n = 62.6\times10^{-6} W \times 580\times10^{-12} s \times 14 = 508\ fJ / Operation$$

$$f = 1/(n\times t_p) = 1/(14\times580\times10^{-12} s) = 20\ MHz$$

The results is summarized in **Figure S9.** The energy consumption increases linear with states number of DW-MTJ based synapse and activation function generator. Therefore, it's a tradeoff between recognition accuracy of the final network and energy consumption. However, recognition accuracy is fast to saturation with the increasing bit number of the sigmoid activation function generator. In most case, a 3-bit (8-state) sigmoid activation function generator is enough for dinky accuracy[14,18].

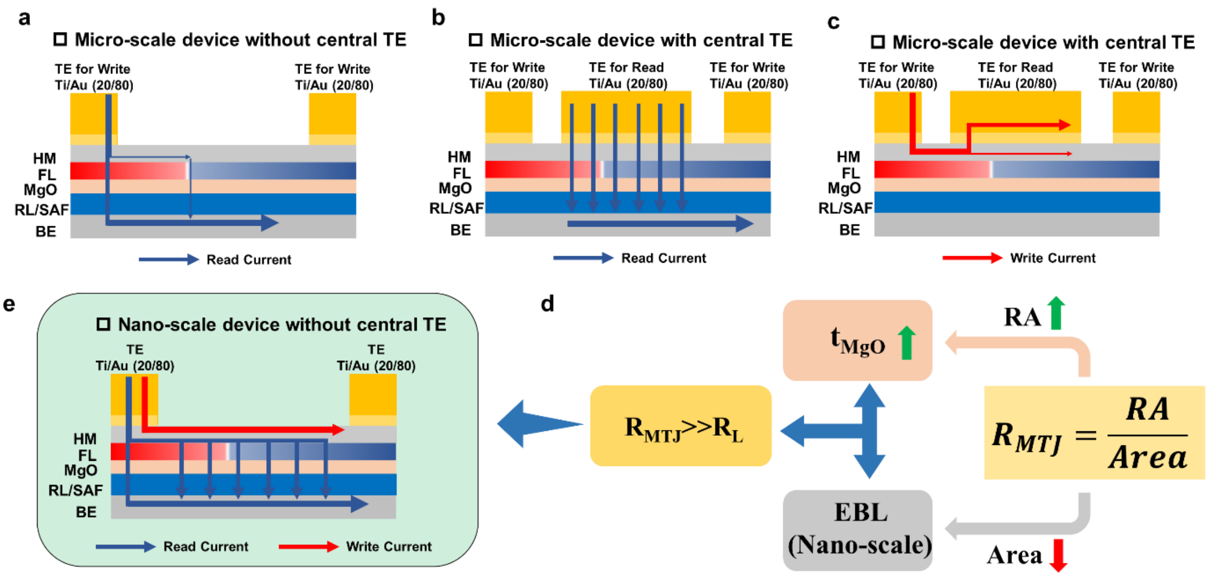

Figure S10 a-c Dilemma of simultaneous electrical write and detection in the present DW-MTJ based synapse and neuron. **d-e** Solution to tackle dilemma by shrinking device and increasement of RA to increase junction resistance.

As shown in **Figures S10 a-c**, the simultaneous electrical write and detection is forbad in present micro-scale DW-MTJ based multistate device, owing to non-trivial shunting effect of write current resulted by the central top electrode (Ti/Au 20nm /80 nm) which is crucial for multi-state read for large device size with small junction resistance (~few Ω). However, when the device scaling down to nano-scale or increase the RA as shown in **Figure S10d**, the magnetoresistance outweighs longitudinal resistance of write channel in device which eliminate the demand of central top electrode for read operation. As shown in **Figure S10e**, the read current injected from left top electrode flows along write channel and then flows down to bottom electrode at each section[19]. While the write current mainly flows between left and right top electrode to maximum effective write current. Besides, device scaling is also beneficial to energy consumption owing to reduced channel resistance and switching current.

Supplementary References

1 Chen, T. Y., Erickson, M. J., Crowell, P. A. & Leighton, C. Surface Roughness Dominated Pinning Mechanism of Magnetic Vortices in Soft Ferromagnetic Films. *Phys. Rev. Lett.* **109**, 097202 (2012).

2 Van de Wiele, B., Laurson, L. & Durin, G. Effect of disorder on transverse domain wall dynamics in magnetic nanostrips. *Phys. Rev. B* **86**, 144415 (2012).

3 Albert, M., Franchin, M., Fischbacher, T., Meier, G. & Fangohr, H. Domain wall motion in

perpendicular anisotropy nanowires with edge roughness. *J. Phys.: Condens. Matter* **24**, 024219 (2012).

4 Nakatani, Y., Thiaville, A. & Miltat, J. Faster magnetic walls in rough wires. *Nat. Mater.* **2**, 521-523 (2003).

5 Vansteenkiste, A. *et al.* The design and verification of MuMax3. *AIP Adv.* **4** (2014).

6 Zhao, X. *et al.* Field-Free Switching of a Spin-Orbit-Torque Device Through Interlayer-Coupling-Induced Domain Walls. *Phys. Rev. Appl.* **13**, 044074 (2020).

7 Lee, S.-W. & Lee, K.-J. Emerging Three-Terminal Magnetic Memory Devices. *Proc. IEEE* **104**, 1831-1843 (2016).

8 Landesman, J.-P. *et al.* Mechanical stress in InP and GaAs ridges formed by reactive ion etching. *J. Appl. Phys.* **128**, 225705 (2020).

9 Wang, C. *et al.* Compact Model of Dzyaloshinskii Domain Wall Motion-Based MTJ for Spin Neural Networks. *IEEE Trans. Electron Devices* **67**, 2621-2626 (2020).

10 Martinez, E., Emori, S., Perez, N., Torres, L. & Beach, G. S. D. Current-driven dynamics of Dzyaloshinskii domain walls in the presence of in-plane fields: Full micromagnetic and one-dimensional analysis. *J. Appl. Phys.* **115**, 213909 (2014).

11 Siddiqui, S. A. *et al.* Magnetic Domain Wall Based Synaptic and Activation Function Generator for Neuromorphic Accelerators. *Nano Lett.* **20**, 1033-1040 (2020).

12 Yang, S. *et al.* Integrated neuromorphic computing networks by artificial spin synapses and spin neurons. *NPG Asia Mater.* **13** (2021).

13 Amin, M. H., Elbtity, M., Mohammadi, M. & Zand, R. in *Proceedings of the Great Lakes Symposium on VLSI 2022,* 319–323 (Association for Computing Machinery, Irvine, CA, USA 2022).

14 He, Z. & Fan, D. A tunable magnetic skyrmion neuron cluster for energy efficient artificial neural network. In *Design, Automation & Test in Europe Conference & Exhibition (DATE), 2017.* 350-355.

15 Xing, S. & Wu, C. Implementation of A Neuron Using Sigmoid Activation Function with CMOS. In *2020 IEEE 5th International Conference on Integrated Circuits and Microsystems (ICICM).* 201-204.

16 Baccelli, G., Stathis, D., Hemani, A. & Martina, M. NACU: A Non-Linear Arithmetic Unit for Neural Networks. In *2020 57th ACM/IEEE Design Automation Conference (DAC).* 1-6.

17 Shamsi, J. *et al.* Hyperbolic tangent passive resistive-type neuron. In *2015 IEEE International Symposium on Circuits and Systems (ISCAS).* 581-584.

18 Ramasubramanian, S. G., Venkatesan, R., Sharad, M., Roy, K. & Raghunathan, A. in *Proceedings of the 2014 international symposium on Low power electronics and design* 15-20 (2014).

19 Kumar, D. *et al.* Ultralow Energy Domain Wall Device for Spin-Based Neuromorphic Computing. *ACS Nano* **17**, 6261-6274 (2023).
