# Supplementary material for: Domain wall magnetic tunnel junction-based artificial synapses and neurons for all-spin neuromorphic hardware
Source: Nat Commun. 2024 May 28;15:4534. doi: 10.1038/s41467-024-48631-4 (PMC11133408; doi:10.1038/s41467-024-48631-4)
Supplement: Supplementary file 3 — Description of Additional Supplementary Files [file 41467_2024_48631_MOESM3_ESM.pdf]

Description of Additional Supplementary Files

Supplementary Movie 1 Animation of the set and reset processes of the proposed sigmoid activation function generator from the micromagnetic simulation.

Supplementary Movie 2 State-by-state MOKE imaging of set procedure as a function of B-field pulse number.

Supplementary Movie 3 State-by-state MOKE imaging of set procedure as a function of B-field pulse amplitude.

Supplementary Movie 4 State-by-state MOKE imaging of set procedure of synaptic device as a function of pulsed J_e.

Supplementary Movie 5 State-by-state bi-directional MOKE imaging of set/reset procedure of sigmoid neuron device as a function of 10 μs pulsed J_e.

Supplementary Movie 6 State-by-state bi-directional MOKE imaging of set/reset procedure of sigmoid neuron device as a function of 50 μs pulsed J_e.

Supplementary Movie 7 Visualization of experimental verification of spin neuron circuit with implementation leveraged by DW-MTJs synaptic and sigmoidal hardware.
